# Choice Experiment Assessment of Consumer Preferences for Yogurt Products Attributes: Evidence from Taiwan

**DOI:** 10.3390/nu14173523

**Published:** 2022-08-26

**Authors:** Min-Yen Chang, Chien-Cheng Huang, Ying-Chi Du, Han-Shen Chen

**Affiliations:** 1 Department of Accounting, Jiaxing University, Jiaxing 314001, China; 2 Department of Health Industry Technology Management, Chung Shan Medical University, Taichung City 40201, Taiwan; 3 Division of Forest Protection, Taiwan Forestry Research Institute, 53 Nan-Hai Road, Taipei 10066, Taiwan; 4 Department of Medical Management, Chung Shan Medical University Hospital, No. 110, Sec. 1, Jianguo N. Rd., Taichung City 40201, Taiwan

**Keywords:** health food certification labels, consumer behavior, food choice, food and health, willingness to pay

## Abstract

Previous studies on consumer yogurt preferences have mainly focused on added sugar, nutrient content, and health claims, leaving several knowledge gaps that should be filled through in-depth research. In this study, a more complete multi-attribute preference model was developed using the number of probiotic types, type of milk source, presence of edible gels (GEL), and usage of health food labels as the main yogurt attributes. A choice experiment (CE) was then conducted to investigate the relationship between multiple attribute preferences and willingness-to-pay (WTP). A total of 435 valid questionnaires were collected by the convenience sampling method. The results show that (1) respondents highly value the health food label (HEA), followed by the number of probiotic types (PRO); (2) the highest WTP in the conditional logit (CL) model was New Taiwan Dollar (NTD) (USD 10.5 for HEA, and the lowest was NTD 1.0 for 100% milk powder (MLK_2_); (3) in the random-parameter logit (RPL) model, the highest WTP was NTD 14.6 for HEA, and the lowest was NTD 2.8 for GEL; (4) the most preferred attribute combination of yogurt was “8 or more probiotic types”, “a blend of raw milk and milk powder”, “the absence of edible gels”, “the presence of a health food label”, and “a price premium of NTD 6–10”; (5) married respondents with children were more willing to pay extra for yogurt products with a higher number of probiotic types and a health food label. The results may help the food industry understand and pay attention to consumer needs, which will, in turn, provide a reference for future product development and marketing strategies.

## 1. Introduction

As consumers are increasingly becoming health-conscious, the demand for healthier foods is also increasing. Among them, yogurt is globally recognized as a healthy diet option that provides easy access to a wide range of nutrients such as proteins, minerals, and vitamins, as well as probiotics [1]. Studies have confirmed that yogurt has positive effects on treating diseases including obesity, allergies, intestinal tract inflammation, colon cancer, cardiovascular disease, and *Helicobacter pylori* infection [2,3,4,5,6,7], which helps to improve human health and reduce the risk of disease [8,9]. According to an international market research firm, the global yogurt market was worth approximately NTD 324.8 billion (USD 10.84 billion) in 2020 and is growing at an annual rate of 4.5% [10], while fermented milk sales in Taiwan have increased from NTD 3.7 billion (USD 120 million) in 2011 to NTD 4.98 billion (USD 170 million) in 2021, a growth rate of 34.6% [11].

As food choice is a complex process that is influenced by personal, environmental, and food-related factors and their interactions [12,13], consumers may have different consumption purposes even for the same product. Consumers often choose various yogurt flavors to satisfy diverse needs [14], including their favorite flavors [15,16]. Many yogurt manufacturers have multiple product lines of varying quality and price, with each line offering multiple flavors at the same price and introducing new flavors from time to time [17]. Therefore, consumer preferences for certain food attributes are important for food producers and processors as well as policymakers to know [18,19]. Roininen et al. [20] revealed that the consumers’ motivation to consume products is one of the best predictors of consumer choice behavior.

Existing studies on yogurt products have mostly focused on added sugar, nutritional content, and health claims [21,22]. Yogurt varies in flavor, texture, and appearance depending on the fermenting strain, milk source, and formulation that is used. Different probiotic types produce different active metabolites, and no consensus has been reached on whether the health benefits of using more probiotic types in yogurt are more than they are when using a single type of probiotic [23,24,25,26]. The yogurt industry in Taiwan generally uses a blend of raw milk and milk powder as the primary milk source, but this may affect consumer preference for yogurt, as consumers maybe have negative impressions of milk powder safety risk and the nutrient loss caused by cumbersome processing [27,28,29].

In addition, with the rise in health consciousness, consumers have become more demanding in terms of food safety, expecting products to be made from more natural and safe ingredients with fewer food additives. Most stabilizers are food additives, some of which have been demonstrated to have effects in the brain leading to memory, behavioral, cognitive, and locomotive dysfunctions [30]. A growing number of studies have shown that food additives may have negative long-term health effects on humans [31,32,33,34]. Most food additives, such as stabilizers, are often added to the industrial production of yogurt to improve its texture and taste. In Taiwan, pectin, guar gum, and locust bean gum, commonly used as stabilizers in yogurt, were officially regulated as food additives instead of food raw materials on 1 July 2022, and thus deserve further research in this study.

As the yogurt business opportunity continues to expand, many products with health claims have emerged in the market, making consumer decisions on choosing healthy foods difficult, seriously compromising consumer health and rights, and making many consumers skeptical of industry claims, leading to demands for clear and reliable product information [35,36]. As a result, consumers pay attention to certification labels that are issued by the government or credible private organizations when purchasing healthful products [37,38]. In Taiwan, the health food label (commonly known as the “Little Green Man” label) has been used since 2000, and foods that have been scientifically verified for safety and health benefits and granted the food label can be considered healthy [39]. However, despite the high consumer interest in perceived health effects and associated health benefits [40,41], most of the existing studies focus on the impact of organic labeling, nutrition labeling, and food safety certification on consumer behavior [42,43,44,45,46]. Given that consumption is susceptible to contextual factors, we expect that the health food label may affect the consumers’ yogurt consumption. Therefore, this study aims to develop a more comprehensive attribute preference model using the number of probiotic types, the milk source, the addition of edible gels, and the use of health food labels as predictor variables.

In experimental economics, the choice experiment (CE) method is a consumer demand analysis method with a well-tested basis in random utility theory that explains the consumers’ behavioral responses [47]. Since the CE method allows the prediction of consumer preference, consumer behavior, and consumer willingness to pay (WTP) [48], it has been widely applied in studies related to food and beverage, including health food [49,50,51]. In addition, consumers’ perceptions and attitudes (e.g., preference) towards the quality and economic value of products or services are often important factors affecting consumer decisions [52], which in turn influence the price premium or maximum price that consumers are willing to pay for a product or service, which is also known as the WTP [53,54].

De-Magistris and Lopéz-Galán [55] used the CE method to examine Spanish consumers’ WTP for cheese and found that respondents were willing to pay a positive premium for low-fat cheese (€ 0.538/100 g) and low-fat, low-salt cheese packs (€ 1.15/100 g), while there was no significant change in WTP for low-salt cheese. Maruyama et al. [50] used the CE method to investigate consumer preferences and purchase prices for yogurts with stabilizers in the US and found that respondents were willing to pay an additional USD 2.54–3.53 for yogurts without stabilizers. Moro et al. [56] used the CE method to investigate Italian consumers’ preferences and WTP for a hypothetical yogurt (assuming the presence of catechin and probiotics as additional ingredients) and found that consumers had a higher WTP a price premium for catechin (€0.38/can) than for probiotics (€ 0.21/can). Livingstone et al. [57] used the CE method to examine the dietary preferences and behaviors of specific ethnic groups in Australia. They found that adults that were aged 18–30 valued nutritional content most highly, followed by cost, taste, familiarity, and meal preparation time, with the dietary preference of female respondents with a higher education level being more affected by nutritional content, taste, and familiarity.

The primary objective of the present study is to examine the effect of preference and health-related consumption purpose (health-oriented consumption motivation) and their interaction on consumer food choice. It analyzes consumer preferences and WTP concerning the consumption of yogurt. The study was conducted on a representative sample of Taiwanese families. First, consumer preferences were investigated through a CE method to validate the origins of the behavior linked to buying yogurt. Second, the drivers of that consumption and the WTP were identified using random utility models to measure the rank of each attribute in shaping consumer preferences. The results may provide useful information for producers, processors, and wholesalers and new insights for policymakers to help them design strategies to promote healthy food choices.

## 2. Materials and Methods

### 2.1. Survey Design

This study focused on the Taiwanese market, where, in terms of fat content, commercially available yogurt mainly comes in two forms: full-fat yogurt and low-fat yogurt; the milk fat content of yogurt can be labeled on food labels at the discretion of food manufacturers [58]. Full-fat yogurt is not labeled as full-fat on the product packaging, while low-fat yogurt is commonly labeled as low-fat on the product packaging. The term low-fat is often used in healthy diet claims, and Taiwanese consumers may be influenced by the product attributes of fat content when purchasing dairy products [15]. In this study, all attributes except price are health attributes. In order to avoid the possible experimental bias caused by the term low-fat in the questionnaire survey, the milk fat content of the hypothetical product was set as full-fat in this study. Yogurts are currently available in four sizes: small (about 200 mL), medium (about 500 mL), large (about 900 mL), and extra-large (about 1700 mL). In this study, all investigated yogurt products were medium in size. Moreover, the following yogurt product attributes were considered in this study: the number of probiotic types, milk source, edible gels, health food label, and price. Table 1 provides the attributes in great detail.

From the above attributes and levels, 144 (3 × 3 × 2 × 2 × 4) combinations were obtained. Since a large number of combinations would lead to difficulties in questionnaire filling and thus a data bias, an orthogonal experimental design was performed in SPSS to further screen combinations. After eliminating unreasonable combinations, one combination representing the status quo and two alterative combinations with randomized values were used to form a single collection of combinations for a single version of the questionnaire, totaling six versions through pairing.

The first part of the formal questionnaire consisted of four parts. The first part was to understand the frequency, motivation, and channels of consumption of yogurt; the second part was to gauge the respondents’ knowledge and valuation of various yogurt attributes (all questions in this part were scored on a 5-point Likert scale by the respondents based on their knowledge and the actual situation, with a score of 1 meaning “strongly disagree” and a score of 5 “strongly agree”). The third part was to gauge the respondents’ preference for yogurt attributes, where each collection of choices consisted of three combinations of choices, with one combination designed for the status quo and two designed through screening (Table 2). The respondents were asked to respond to questions on their preferences. The fourth part investigated the respondents’ socio-economic background, including gender, age, marital status, education level, average monthly income, and physical health. The body mass index (BMI) was one of the two health indicators that were investigated in this study. According to the Health Promotion Administration of Taiwan’s Ministry of Health and Welfare in 2021 [59], adults over 18 years of age in Taiwan are classified into four categories by BMI: underweight (BMI < 18.5), healthy weight (18.5 ≦ BMI < 24), overweight (24 ≦ BMI < 27), and obese (BMI ≧ 27). The second indicator was adult waist circumference: (1) for male adults, waist circumference < 90 cm and ≧90 indicates healthy and obese individuals, respectively; (2) for female adults, the two thresholds become <80 cm and ≧80 cm, respectively. Waist circumference is commonly used as a simple measure to determine the risk of metabolic syndrome and cardiovascular disease.

### 2.2. Choice Analysis: Conceptual Framework and Statistical Model

Choice experiments are based on Lancaster’s characteristics and random utility theories. The theories assume that the utility an individual derives from a product depends on its individual characteristics and the unobserved (stochastic) components [60]. The CE method can create hypothetical market goods or services to investigate consumers’ multiple attribute preferences, WTP prices, and socio-demographic interrelationships [61]. CE surveys used stated preferences (SP) as the primary method. SP do not need real market conditions or actual consumer behavior but directly uses the pre-set attributes and levels in the study to conduct questionnaire interviews, allowing for the design, analysis, and application of survey experiments for consumer preference prediction.

The CE method is also commonly used to investigate the impact of food labeling on consumers’ purchasing decisions, as food labels reveal more information about food products, including their contents, capacity specifications, precautions, nutritional content, relevant regulations, and certifications. Consumers use food labels to understand food characteristics, safety, and health information before making purchase decisions. Van den Akker et al. [45] investigated the impact of the new front-of-package (FOP) nutrition label on consumers’ choice of healthy diets in the Netherlands and found that the new nutrition labels were better than the previous ones, which is beneficial for promoting new food policies. Wilde et al. [46] used the CE method to investigate the interaction between hypothetical products and actual product labels and found that U.S. respondents were prone to cognitive bias toward food labels provided by food manufacturers; the results of the study may help the government to amend laws to urge food manufacturers to adjust their food labels. Kim et al. [16] set yogurt image, taste description, probiotics claim, and nutritional information as preferred attributes on the package label, with each attribute having two levels (health vs. non-health). They then used the CE method and a remote eye-tracker to record and analyze cognitive reflection test (CRT) data of Korean female consumers to further analyze the relationship between health-related consumption and purchase decisions.

In the present study, we used a random-parameter logit (RPL) model, which assumes that respondents had heterogeneous preferences for yogurt attributes, and a conditional logit (CL) model, which assumes that respondents have the same preference for yogurt attributes.

The utility function of the *i*-th respondent for the *j*-th option of product can be described by Equation (1):

(1) $$U_{ij}=V_{ij}+\varepsilon_{ij}=V_{ij}\left(W_{j}\right)+\varepsilon_{ij}$$

where $U_{ij}$: the utility of the *i*-th respondent for the *j*-th product attribute combination;

$V_{ij}$: an observable component, representing the observed utility of the *i*-th respondent for the *j*-th product attribute combination;

$\varepsilon_{ij}$: an unobservable component, which is the random error;

$W_{j}$: the *j*-th product attribute combination.

Assuming that the indirect utility function of the *i*-th respondent for $W_{j}$ can be described by a linear additive model (LAM), and denoting the corresponding price attribute as *P_j_*, Equation (1) can be expressed as:

(2) $$U_{ij}=V_{ij}+\varepsilon_{ij}=V_{ij}\left(W_{j}\right)+\varepsilon_{ij}=\overset{k}{\underset{k=1}{\sum }}\alpha_{k}X_{jk}+\beta P_{j}+\varepsilon_{ij}$$

where $X_{jk}$: the *k*-th non-price attribute of the *j*-th product in $W_{j}$;

$P_{j}$: the price attribute of the *j*-th product;

$\alpha_{k}$: the value of product attribute variable *X_jk_*;

β: the value of price attribute *P_j_*.

The present study aimed to explore the influence of the socio-economic background of the interviewed group on product attribute preferences. According to Burton et al. [62], when estimating the indirect utility function, the interaction between product attribute combination $W_{j}$ and the socio-economic background of the respondents should be considered. Therefore, Equation (2) is rewritten as Equation (3) and further as Equation (4), which allows the relationship between preferred attributes and WTP to be readily observed.

$$U_{ij}=\overset{k}{\underset{k=1}{\sum }}\alpha_{k}X_{jk}+\beta P_{j}+\varepsilon_{ij}$$

(3) $$=\overset{k}{\underset{k=1}{\sum }}\alpha_{k}X_{jk}+\sum_{k=1}^{K}\sum_{q=1}^{Q}\gamma_{kq}X_{jk}Z_{iq}\beta P_{j}+\varepsilon_{ij}$$

(4) $$=\sum_{k=1}^{K}\alpha_{k}X_{jk}+\beta P_{j}+\sum_{k=1}^{K}\sum_{q=1}^{Q}\gamma_{kq}X_{jk}Z_{iq}+\sum_{k=1}^{K}\sum_{q=1}^{Q}\gamma_{pq}P_{j}Z_{iq}+\varepsilon_{ij}$$

Here, $\gamma_{kq}$: the interaction coefficient of a product attribute and a socio-economic background;

$\gamma_{pq}$: interaction coefficient of the price attribute and socio-economic background;

$Z_{iq}$: the socio-economic background of the *i*-th respondent.

To understand the respondents’ preferences for yogurt attributes, their socio-economic background and attitude were included as alternative-specific constants (ASCs) for attributes and then incorporated into the utility function according to Baskaran et al. [63]. Accordingly, the Equation now becomes:

(5) $$V_{ij}=ASC_{j}+\underset{k}{\sum }\;B_{k}X_{ijk}+\underset{m}{\sum }\;\theta_{jm}ASC_{j}\times S_{mi}+\underset{n}{\sum }\;\delta_{kn}X_{ijk}\times S_{ni}$$

where $V_{ij}$: an observable component, representing the utility of the *i*-th respondent for the *j*-th product;

$ASC_{j}$: the *j*-th product-specific constant;

$X_{ijk}$: the *k*-th attribute of the *j*-th product in $W_{j}$;

$\theta_{jm}$: a vector of the interaction coefficients between ASC and the *m*-th socio-economic characteristic of the *i*-th respondent;

$S_{mi}$: the interaction coefficient between ASC and the *m*-th socio-economic characteristic of the *i*-th respondent;

$\delta_{kn}$: a vector of the interaction coefficients between the *k*-th attribute and the *n*-th socio-economic characteristic of the *i*-th respondent;

$S_{ni}$: the interaction coefficient between the *k*-th attribute and the *n*-th socio-economic characteristic of the *i*-th respondent.

To measure the WTP price for a product attribute, Equation (2) is fully differentiated and, assuming that the utility remains the same, $dU_{ij}=0$ yields Equation (6):

(6) $$dU_{ij}=\overset{k}{\underset{k=1}{\sum }}\alpha_{k}dX_{jk}+\beta dP_{j}=0$$

Letting other attributes remain unchanged ($dX_{j1}=dX_{j2}=\cdots =dX_{jk-1}=0)$, the consumer’s WTP for $X_{jk}$, the *k*-th attribute of the *j*-th product, can be derived as follows:

(7) $$WTP_{k}=-\frac{dP_{j}}{dX_{jk}}=-\frac{\alpha_{k}}{\beta }$$

where $X_{jk}$: the *k*-th attribute of the *j*-th product in $W_{j}$;

$\alpha_{k}$: the value of product attribute variable *X_jk_*;

β: the value of price attribute *P_j_*.

## 3. Results and Discussion

### 3.1. Sample Size and Composition

In this study, we used a convenience sampling method to present a questionnaire, face-to-face, in supermarkets and convenience stores. First, the study conducted a pre-test questionnaire, with the aim of understanding consumers’ overall consumption preferences and WTP for yogurt. The questionnaires were issued from 25 November 2021 to 5 December 2021 to consumers who had purchased yogurt in the last 60 days. One hundred and five questionnaires were issued, out of which eighty-seven were valid, and the effective questionnaire recovery rate was 82.9%. The formal questionnaire was distributed from 13 January 2022 to 15 March 2022, targeting consumers who had purchased yogurt in the last 60 days. A total of 550 questionnaires were issued. After factoring out invalid questionnaires, a total of 435 valid questionnaires were obtained, representing a 79.1% questionnaire recovery rate, and their socioeconomic backgrounds are shown in Table 3.

Female respondents (60.9%) outnumbered male respondents (39.1%), which was consistent with the fact that women are the main purchasers in most households [64,65]. For age distribution, those aged 30–39 years (29.7%) accounted for the largest fraction, followed by the 40–49 years group (23.4%), 50–59 years (19.3%), 18–29 years (18.6%), and then ≥60 years (9.0%). Married and unmarried respondents accounted for 57.9% (10.3% without children and 47.6% with children) and 42.1%, respectively. Regarding education level, the majority had university or junior college education (61.6%), while junior high school (or lower) and doctorate education accounted for less than 3%. The average personal monthly income was mainly in the range of NTD 20,001–40,000 (33.1%) and NTD 40,001–60,000 (29.0%).

As far as personal health data were concerned, 42.1% of the respondents were found to have a healthy weight (18.5 ≤ BMI < 24), followed by 29.2% overweight (24 ≤ BMI < 27), and 10.6% were obese (BMI ≧ 27), with the latter two totaling 39.8%. According to the statistics from the Accounting Office of the Taiwan Executive Yuan (2021) [66], in 2013–2016, the percentage of overweight and obese adults, according to their BMI, that were over 19 years old in Taiwan accounted for 52.1% of the male adult population, representing an increase of 0.6% when compared with the previous survey (2005–2008) and an increase of 18.7% in 1993–1996. In 2013–2016, the percentage of overweight and obese adults, according to their BMI, that were over 19 years old in Taiwan accounted for 37.4% of the female adult population, representing an increase of 0.5% when compared with the previous survey (2005–2008), and an increase of 4.4% over in 1993–1996. The survey of the BMI data shows that Taiwanese adults are gradually becoming overweight and obese. The results of this study are consistent with the long-term trend and results of the BMI survey conducted by the Directorate-General of Budget from the Accounting and Statistics departments of the Executive Yuan of Taiwan.

The Survey of respondents’ experiences in purchasing yogurt are shown in Table 4. Consumption frequency was defined as the number of times yogurt was purchased per month, with 2–3 times as the highest frequency (40.5%), followed by once as the second highest frequency (38.4%). It is speculated that the reason for the low consumption frequency is that for Taiwanese consumers, the average availability of milk is only 0.6 servings per person per day due to Taiwan’s limited milk production and high dependence on imports [67]. Furthermore, according to a survey by Numbeo [68], the most expensive price for a liter of milk is in Lebanon at USD 4.80 per liter, followed by Taiwan at USD 3.10, and Hong Kong at USD 3.04. The consumption channels were mainly supermarkets (43.2%) and convenience stores (33.8%). The main consumption motives were to improve health (41.6%) and to supplement nutrition (23.9%), which together accounted for 65.5%, suggesting that the majority of respondents consumed yogurt for health purposes [69,70].

### 3.2. Knowledge and Valuing of Each Attribute

The knowledge and valuing of each attribute were designed using a 5-point Likert scale ranging from 1 = strongly disagree, to 5 = strongly agree, for the measurement of the inquired respondents about the extent to which they agreed with each item. The Survey of the respondents’ knowledge of yogurt product information are shown in Table 5. The results show that respondents’ knowledge of each attribute was the lowest for “the usefulness of edible gels” (2.70), followed by “the difference between raw milk and milk powder” (3.16), and both scores were lower than the average score, implying that the respondents had a moderate level of knowledge of yogurt product information.

The Survey of respondents’ attention to yogurt product information are shown in Table 6. The degree to which the respondents valued each attribute was highest for “presence or absence of health food label” (4.33), followed by “the number of probiotic types” (4.09), “product price” (3.89), “raw milk or milk powder as raw material” (3.88), and then “presence or absence of edible gels” (3.60). The results revealed that the respondents most highly valued the presence or absence of a health food label among all investigated attributes of yogurt products. Although yogurt has been recognized globally as a component of a healthy diet because it helps to improve health and supplement daily nutrition, consumers still value a credible certification label in them making their decisions. This is consistent with Kaczorowska et al. [37], who found that credible food certifications can help consumers to select healthier and safer products. FOP information can guide consumers to choose healthier yogurt products when they are making their purchase decisions. Therefore, FOP information may be leveraged to help consumers learn more about healthy product attributes, in turn creating a market niche. Consumption utility will significantly improve if guidance is provided through easily understandable, necessary information.

### 3.3. Consumer Preferences of Yogurt Attribute Combinations

The most preferred attribute combination of yogurt was “8 or more probiotic types” plus “a blend of raw milk and milk powder as the milk source” plus “the absence of edible gels” plus “the presence of health food label” plus “a price premium of NTD 6–10” (21.7%), followed by the combination of “2–4 probiotic types” plus “100% milk powder as the milk source” plus “the presence of edible gels” plus “the presence of health food label” plus “a price premium of NTD 0” (19.2%). The least preferred attribute combination was “2–4 probiotic types” plus “100% raw milk as the milk source” plus “the absence of edible gels” plus “the absence of health food label” plus “a price premium of NTD 1–5” (3.1%).

### 3.4. Results of CL and RPL Analysis

The coefficient of each attribute variable in the CL and RPL models was calculated using NLOGIT 4.0; the empirical estimates are shown in Table 7. The coefficient for “maintaining the status quo” (ASC) was negative in both the CL and RPL models, indicating that respondents did not prefer to keep the current attribute combination.

In the CL and RPL models, the results show that consumers cared more about PRO_1_ and HEA for yogurt products than they did for other attributes. It is consistent with Bimbo et al. [70], that some consumers prefer probiotics-claiming dairy products. In addition, when consumers purchase yogurt products, they generally identify the product content through the FOP information, including the ingredients, nutritional content, and certification label. FOP information can guide consumers to purchase healthier products [71]. Therefore, enhancing consumer knowledge of FOP information can help them to choose products with fewer food additives [72,73]. Based on the results of this study, the number of probiotic types and the presence of a health food label are very important for their effects on the consumers’ purchasing decisions of yogurt products. Thus, the food industry needs to pay more attention to the enhancement of the number of probiotic types and the health food label.

The coefficients derived from the utility Function (1) were substituted into the theoretical model (7) to calculate the WTP of the respondents. In the CL model, the WTP associated with each attribute was as follows: NTD 5.5 (PRO_1_, for 5–7 probiotic types), NTD 9.7 (PRO_2_, for eight and more probiotic types), NTD 3.6 (MLK_1_, for 100% raw milk), NTD 1.0 (MLK_2_, for 100% milk powder), NTD 1.8 (GEL, for the absence of edible gels), and NTD 10.5 (HEA, for the presence of a health food label). In the RPL model, the WTP associated with each attribute was as follows: NTD 3.7 (PRO_1_, for 5–7 probiotic types), NTD 6.3 (PRO_2_, for eight and more probiotic types), NTD 3.1 (MLK_1_, for 100% raw milk), NTD 3.9 (MLK_2_, for 100% milk powder), NTD 2.8 (GEL, for the absence of edible gels), and NTD 14.6 (HEA, for the presence of health food label).

### 3.5. Respondents’ Differences in Yogurt Attribute WTP with Respect to Socio-Economic Background

As shown by the RPL model results, the coefficients of the two attribute variables, PRO_2_ and HEA, were random, suggesting that it was necessary to examine how the socio-economic backgrounds of respondents affected the WTP associated with each of the two attribute variables. As shown in Table 8, the WTP associated with PRO_2_ varied significantly with education level and marital status. Respondents who had a college education and were married with children were more willing to pay extra for yogurt products containing more probiotic types. This was consistent with the findings of Vatanparast et al. [74] that consumers with a college education and who were married with children are more willing to purchase healthy probiotic yogurts.

The WTP that were associated with HEA varied significantly with age, marital status, and average personal monthly income. In particular, those that were aged 40–49 years, married (with children), and with an average personal monthly income of NTD 40,001–60,000 were willing to pay extra for yogurt products with a health food label. According to Van Loo et al. [65], high-income married families with children are willing to purchase products with a health food label that claims that it is healthier. In contrast, consumers that were aged 18–29 years, unmarried, and with an average personal monthly income of NTD 20,001–40,000, in the present study were less likely to pay extra for a health food label.

## 4. Discussion

As shown above, WTP was highest for yogurts that showed the presence of a health food label (HEA) in both the CL and RPL models, suggesting that respondents were willing to pay more for products with a health food label, which was consistent with the finding by Wang et al. [75] that food certification labeling affects consumer WTP. The second highest WTP was for yogurts with the presence of eight or more probiotic types (PRO_2_), indicating that respondents were willing to pay more for probiotics-claiming products, which was consistent with Bimbo et al. [70] that some consumers prefer probiotics-claiming dairy products. So far, seven probiotic types have been claimed to be in commercially available yogurt products, suggesting that consumer demand is driving food producers to develop new products, which is in line with the increasing global trend of probiotics, with a 7% annual growth rate and a total market of USD 45.6 billion in 2017 [76].

Studies have shown that multiple strains of probiotics are better than single strains of probiotics in treating human diseases and maintaining physical health [24,77,78]. However, there are also other studies that indicate that only a small number of multi-strain probiotics are more beneficial to humans than single-strain probiotics; more clinical trials are needed to prove this [23,25,26].

Regarding the WTP for the presence of edible gels in yogurts, in the CL model, the NTD was 1.8, while the NTD was 2.8 in the RPL model, suggesting that the respondents had lower preference for products without edible gels, presumably due to their low level of knowledge of edible gels. When consumers purchase yogurt products, they generally identify the product’s content through the FOP information, including its brand name, name, ingredients, date of manufacture, nutritional content, and certification label. FOP information can guide consumers to purchase healthier products [71]. Therefore, enhancing consumer knowledge of FOP information can help them to choose products with fewer food additives [72,73].

In both the CL and RPL models, the WTP for the presence of eight or more probiotic types (PRO_2_) was second only to the WTP associated with HEA, indicating that respondents were willing to pay extra for probiotic-claiming products, which is in line with the finding of Bimbo et al. [70]. The WTP for the absence of edible gels was evidenced by the NTD value of 1.8 in the CL model, and an NTD of 1.8 in the RPL model, suggesting that the presence edible gels did not provide an incentive for respondents to increase their willingness to pay extra, presumably due to their low level of knowledge of edible gels.

According to Cavaliere et al. [79], young Italian consumers have a lower concern for health risks and are therefore less interested in diet-related health claims; conversely, older consumers have a greater concern for health risks, and therefore place more importance on diet-related health claims, leading to the purchase of healthier products. Ballco and De Magistris [69] proved that women that were aged 18–34 years with a university degree are not interested in the health and nutrition claims on yogurt products, which is consistent with the present study.

## 5. Conclusions

### 5.1. Management Implications

As shown by the survey results, the respondents’ knowledge of product information was only at an average level, but after the status quo and meaning of each attribute were explained to the respondents, they placed significantly higher importance on each attribute. In other words, increasing the consumers’ knowledge of product attributes can help them to better understand the importance of product attributes. Meanwhile, FOP information can guide consumers to choose healthier products when they purchase yogurt products. Therefore, FOP information may be leveraged to help consumers to learn more about healthy product attributes, therefore creating a market niche. Consumption utility will be greatly improved by providing this easily understandable, necessary information.

Secondly, the respondents preferred products with a health food label, suggesting that yogurt products with a health food label would be more attractive to consumers. Although yogurt has been recognized, globally, as a component of a healthy diet because it helps to improve health and supplement daily nutrition, consumers still value a credible certification label to help them make their decisions. Therefore, for yogurt products already granted health food labels by a central competent authority in Taiwan, efforts should be made to keep the labels understandable. It is also desirable to apply for the addition or reinstatement of health food labels for more yogurt products. Moreover, it may be possible to consider applying for healthy yogurts to be considered among other Taiwanese products for their 11 kinds of health benefits (such as immune regulation, bone health care, etc.) as these health benefits have been announced to increase the willingness of consumers to purchase.

The empirical results of both the CL and RPL models showed that a healthy food label and the presence of eight or more probiotic types led to the highest and second highest WTP, respectively. An increase in the number of probiotic types led to an increase in WTP and consumer utility. However, whether it is feasible to use more than eight types of probiotics under the practical conditions of industrial yogurt production should be further investigated. Secondly, the lowest or second lowest attribute that WTP was associated with was the absence of edible gels, indicating that consumers are reluctant to increase the additional payment amount for yogurts that do not contain these, likely due to a lack of clear understanding and awareness of the importance of edible gels. However, in line with the international trend, Taiwan officially regulates pectin, guar gum, and locust bean gum, commonly used as edible gels in yogurt, as food additives. Although most consumers are not yet aware of the importance and impact of edible gels, the food industry can consider reducing the use of food additives or using other food ingredients and, at the same time, strengthen the consumers’ knowledge of food additives to help them to choose healthy and safe foods. In addition, the governmental departments can encourage and reward food manufacturers who are committed to developing healthier food.

As shown by the analysis results, consumers who are married with children, have a university or junior college education, and have a high personal income are more likely to purchase healthier yogurt products with a higher number of probiotic types and health food labels for their family members, suggesting that food companies may develop yogurt products with more emphasis on their health features to attract this group of consumers. In contrast, young, unmarried, and average-income consumers placed relatively low importance on the health attributes of products. Therefore, when developing products targeting this group, it may be necessary to explore the food attributes that this group of consumers value.

### 5.2. Research Limitations and Future Research Directions

There are some limitations in the implementation of this survey, and the research framework can only be improved if the research scope can be expanded in the future. The limitations and suggestions are summarized as follows:

1. Only five yogurt product attributes (number of probiotic types, milk source, edible gels, health food label, and price) were included, while there are other yogurt product attributes that could have been included. For example, the fat and protein content of the milk source can be adjusted by adding food processing ingredients such as milk protein concentrates, whey powder, and whey protein, resulting in changes in the texture, flavor, and nutritional composition of yogurt. Meanwhile, differences in consumer understanding and valuing of these food processing ingredients may affect consumer preferences and WTP.
2. The research materials are mainly regarding medium (about 500 mL) yogurts, but there are still small (about 200 mL), large (about 900 mL), and extra-large (about 1700 mL) yogurts in the Taiwan market. It is possible to explore the influence of different specifications of yogurt on consumer preferences and motivation, consumers’ channel choices, as well as further cross-analysis and the relationship between consumers’ social and economic background.
3. The results of this study showed that respondents’ consumption motive was focused on health improvement (41.6%) and nutritional supplementation (23.9%). Therefore, future studies can further explore health improvement-related attributes in depth (e.g., gastrointestinal mediation and prevention of cardiovascular diseases) and nutrition supplementation-related attributes (e.g., calcium and collagen), which should help the food industry to understand consumers’ preferences and WTP to develop healthy yogurts to meet market demand.
4. The latent class model (LCM) may be used in future research to examine whether there is heterogeneity in consumer preferences for yogurt.

## Figures and Tables

**Table 1:** Yogurt attributes and their levels.

Attribute	Level	Variable Name	Variable Value	Expected Sign
Number of probiotic types	2–4 types (current) 5–7 types 8 or more types	PRO_1_	“−1” means “keeping the status quo (2–4 types)” “1” means “5–7 types” “0” means “8 or more types”	+
		PRO_2_	“−1” means “keeping the status quo (2–4 types)” “0” means “5–7 types” “1” means “8 or more types”	+
Milk source	Blend (current) 100% raw milk 100% milk powder	MLK_1_	“−1” means “keeping the status quo (blend)” “1” means “100% raw milk” “0” means “100% milk powder”	+
		MLK_2_	“−1” means “keeping the status quo (blend)” “0” means “100% raw milk” “1” means “100% milk powder”	+
Edible gels	Added Not added	GEL	“−1” means “added” “1” means “not added”	+
Health food label	With a label Without a label	HEA	“−1” means “with a label” “1” means “without a label”	+
Price	Keep the current price NTD 0 Pay a pre-mium NTD 1–5 Pay a premium NTD 6–10 Pay a premium NTD 11–15	FUND	“0” means “NTD 0” “5” means “NTD 1–5” “10” means “NTD 6–10” “15” means “NTD 11–15”	–

Note: NTD: New Taiwan dollar (1 NTD = 0.033 USD).

**Table 2:** Example of choice collection in the questionnaire survey.

	Combination	Alternative 1	Alternative 2	Status Quo
Attribute				
Number of probiotic types		8 or more types 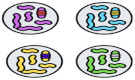	5–7 types 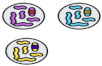	2–4 types 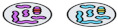
Milk source		Blend (raw milk + milk powder) 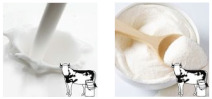	100% raw milk 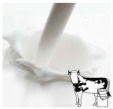	Blend (raw milk + milk powder) 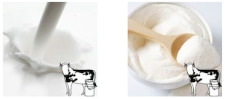
Edible gels		Absence 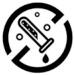	Presence 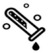	Presence 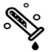
Health food label		Presence 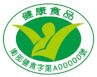	Presence 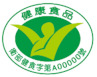	Absence 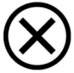
Price (Medium size of about 500 mL)		Additional payment of NTD 6−10 (Original price NTD 49)	Additional payment of NTD 11−15 (Original price NTD 49)	Original price NTD 49
Please check the box		□	□	□

**Table 3:** Demographic information.

Variable	Description	Sample Size	Percentage
Gender	Male	170	39.1%
	Female	265	60.9%
Age (years)	18–29	81	18.6%
	30–39	129	29.7%
	40–49	102	23.4%
	50–59	84	19.3%
	60 or above	39	9.0%
Marriage Status	Unmarried	183	42.1%
	Married (no children)	45	10.3%
	Married (with children)	207	47.6%
Education level	Junior high school or below	10	2.3%
	High school and vocational school	63	14.5%
	University and junior college	268	61.6%
	Master	83	19.1%
	PhD	11	2.5%
Average personal monthly income (NTD)	Up to NTD 20,000	84	19.3%
	20,001–40,000	144	33.1%
	40,001–60,000	126	29.0%
	60,001–80,000	41	9.4%
	80,001–100,000	21	4.8%
	Over NTD 100,001	19	4.4%
BMI (kg/m^2^)	<18.5	31	7.1%
	18.5 ≤ BMI < 24	183	42.1%
	24 ≤ BMI < 27	127	29.2%
	27≤	46	10.6%
	Unknown	48	11.0%
Male waist circumference (cm)	<80	34	20.0%
	80≤ and <90	93	54.7%
	90≤	30	17.6%
	Unknown	13	7.6%
Female waist circumference (cm)	<80	110	41.5%
	80≤ and <90	105	39.6%
	90≤	15	5.7%
	Unknown	35	13.2%

**Table 4:** Respondents’ experiences in purchasing yogurt.

Variable	Description	Sample Size	Percentage
Consumption Frequency (number of purchases per month)	1 times	167	38.4%
	2~3 times	176	40.5%
	4~5 times	52	12.0%
	6 times or more	40	9.2%
Consumption Channel (Most frequently purchased channel)	convenience stores	147	33.8%
	supermarkets	188	43.2%
	hypermarket	89	20.5%
	others	11	2.5%
Consumption Motivation	for no reason	116	26.7%
	to slake hunger	23	5.3%
	to supplement nutrition	104	23.9%
	to improve health	181	41.6%
	others	11	2.5%

**Table 5:** Respondents’ knowledge of yogurt product information.

Description	Respondent Knowledge
How well do you know about the topic of “food labels on outer packaging”?	3.40
How well do you know about the topic of the “benefits of probiotics”?	3.77
How well do you know about the topic of “the difference between raw milk and milk powder”?	3.16
How well do you know about the topic of “the usefulness of edible gels”?	2.70
How well do you know about the topic of “health food labels”?	3.62

**Table 6:** Respondents’ values to yogurt product information.

Description	Respondent Value
How well do you value information about “the number of probiotic types”?	4.09
How well do you value information about “raw milk or milk powder as a raw material”?	3.88
How well do you value information about the “presence or absence of edible gels”?	3.60
How well do you value information about the “presence or absence of health food label”?	4.33
How well do you value information about “product price”?	3.89

**Table 7:** Results of the CL and RPL models.

Attribute and Variable	CL			RPL				
	Coefficient	t-Value	WTP (NTD)	Coefficient	t-Value	Standard Error	t-Value	WTP (NTD)
Status quo (ASC)	−0.304	−1.993 *		−0.721	−0.797 **	0.905	0.831	
Number of probiotic types (PRO_1_)	0.148	1.791 ***	5.5	0.218	2.013 **	0.108	2.255	3.7
Number of probiotic types (PRO_2_)	0.261	0.849 **	9.7	0.371	0.119 ***	0.184	0.071 **	6.3
Milk source (MLK_1_)	0.098	0.217	3.6	−0.184	0.441	0.417	0.583	3.1
Milk source (MLK_2_)	−0.027	−0.129	1.0	−0.228	1.306	0.174	1.137	3.9
Edible gels (GEL)	0.0485	0.533	1.8	0.163	−0.171 *	0.113	0.285	2.8
Health Food Label (HEA)	0.284	6.576 ***	10.5	0.859	3.292 ***	0.261	3.154 ***	14.6
Price (FUND)	0.027	0.012		0.059	1.605	0.037		
Number of attribute combinations	1305			1305				
Log–likelihood ratio	−1134.552			−1027.933				

***, **, and * are significant at 1%, 5%, and 10%, respectively; NTD: New Taiwan dollar (1 NTD = 0.033 USD).

**Table 8:** Respondents’ socio-economic backgrounds and the WTPs associated with selected yogurt attributes.

Socio-Economic Background		Number of Respondents	ASC		PRO_2_		HEA	
			Average Value	t-Value	Average Value	t-Value	Average Value	t-Value
Gender	Male	170	−24,842	2.61	435	1.77	823	3.16
	Female	265	−25,276		551		632	
Age (years)	18–29	81	−21,634	−2.34	320	1.53	735	2.88 *
	30–39	129	−18,955		379		611	
	40–49	102	−22,211		501		853	
	50–59	84	−20,488		325		776	
	60 or above	39	−21,084		319		860	
Marriage Status	Unmarried	183	−22,569	−1.46 *	445	2.44 **	916	2.34 **
	Married (no children)	45	−20,230		410		681	
	Married (with children)	207	−18,790		391		889	
Education level	Junior high school or below	10	−21,320	1.89	544	2.69 *	874	1.08
	High school and vocational school	63	−27,149		339		759	
	University and junior college	268	−20,122		590		697	
	Master	83	−20,456		424		714	
	PhD	11	−21,092		346		749	
Average personal monthly income	Up to NTD 20,000	84	−23,971	−2.47 *	518	3.18	640	2.19 *
	20,001–40,000	144	−18,960		380		715	
	40,001–60,000	126	−20,674		529		857	
	60,001–80,000	41	−21,361		388		667	
	80,001–100,000	21	−22,622		472		464	
	Over NTD 100,001	19	−20,779		596		635	
BMI (kg/m^2^)	<18.5	31	−22,628	4.31	362	2.56	762	1.54
	18.5 ≤ BMI < 24	183	−22,300		290		765	
	24 ≤ BMI < 27	127	−22,440		397		584	
	27≤	46	−21,579		548		862	
	Unknown	48	−24,083		353		704	
Male waist circumference (cm)	<80	34	−19,141	1.19	419	0.95	786	2.43
	80≤ and <90	93	−24,998		465		827	
	90≤	30	−23,667		344		791	
	Unknown	13	−21,100		238		686	
Female waist circumference (cm)	<80	110	−23,653	2.73	313	1.56	828	0.98
	80≤ and <90	105	−22,311		267		695	
	90≤	15	−21,690		329		832	
	Unknown	35	−22,538		347		424	

** and * are significant at 5% and 10%, respectively; ASC: keep the status quo; PRO_2_: number of probiotic types; HEA: health food label; NTD: New Taiwan dollar (1 NTD = 0.033 USD).

## Data Availability

The data that support the findings of this study are available from the corresponding author, H.-S.C., upon reasonable request.
